# Population‐specific genotype x genotype x environment interactions in bacterial disease of early life stages of Pacific oyster larvae

**DOI:** 10.1111/eva.12452

**Published:** 2017-03-09

**Authors:** Carolin C. Wendling, Armin G. Fabritzek, K. Mathias Wegner

**Affiliations:** ^1^ Wadden Sea Station SyltAlfred Wegener Institute, Helmholtz Centre for Polar and Marine ResearchListGermany; ^2^ GEOMAR, Helmholtz Centre for Ocean ResearchKielGermany; ^3^ Department of EcologyInstitute of ZoologyJohannes Gutenberg‐University of MainzMainzGermany

**Keywords:** *Crassostrea gigas*, emerging marine diseases, GxGxE, Pacific oyster, *Vibrio*

## Abstract

The consequences of emerging marine diseases on the evolutionary trajectories of affected host populations in the marine realm are largely unexplored. Evolution in response to natural selection depends on the genetic variation of the traits under selection and the interaction of these traits with the environment (GxE). However, in the case of diseases, pathogen genotypes add another dimension to this interaction. Therefore, the study of disease resistance needs to be extended to the interaction of host genotype, pathogen genotype and environment (GxGxE). In this study, we used a full‐sib breeding design crossing two genetically differentiated populations of the Pacific oyster *Crassostrea gigas* (Thunberg, 1793), to determine the influence of host genotype, pathogen genotype and temperature on disease resistance. Based on a controlled infection experiment on two early life stages, that is, D‐larvae and Pediveliger larvae at elevated and ambient water temperatures, we estimated disease resistance to allopatric and sympatric *Vibrio sp*. by measuring survival and growth within and between genetically differentiated oyster populations. In both populations, survival was higher upon infection with sympatric *Vibrio sp*., indicating that disease resistance has a genetic basis and is dependent on host genotype. In addition, we observed a significant GxGxE effect in D‐larvae, where contrary to expectations, disease resistance was higher at warm than at cold temperatures. Using thermal reaction norms, we could further show that disease resistance is an environment dependent trait with high plasticity, which indicates the potential for a fast acclimatization to changing environmental conditions. These population‐specific reaction norms disappeared in hybrid crosses between both populations which demonstrates that admixture between genetically differentiated populations can influence GxGxE interactions on larger scales.

## 1 Introduction

The potential of species to cope with or adapt to changing environmental conditions has profound effects on populations, species and even ecosystems. Within one generation, responses to environmental change through phenotypic plasticity may reflect the acclimation capacity of individual organisms (Sunday, Crim, Harley, & Hart, 2011). Thus, phenotypic plasticity can help individual organisms to overcome unfavourable conditions on a short timescale. However, environmental changes will impose directional selection on traits relevant for fitness (Gienapp, Teplitsky, Alho, Mills, & Merila, 2008), thereby favoring some genotypes over others, leading to genotype x environment (GxE) interactions. Therefore, standing genetic variation enabling fast cross‐generational responses will determine how populations will evolve, which ultimately allows us to make long‐term predictions about population persistence (Gienapp et al., 2008; Stockwell, Hendry, & Kinnison, 2003).

In the case of disease, another dimension contributes to the GxE interactions of host organisms, namely the genotype of the pathogen. Thus, the evolution of disease resistance does not only depend on the host genotype but also on pathogen genotypes leading to genotype x genotype (GxG) interactions. Many emerging marine diseases are tightly linked to rising temperatures (Burge et al., 2014; Fey et al., 2015; Harvell et al., 1999; Harvell et al., 2002; Lafferty et al., 2015). In particular, ecologically and economically important species, such as seagrasses, corals and oysters, have been frequently subjected to mass mortalities (Harvell et al., 1999). Rising temperatures may increase pathogen proliferation and transmission rate and on the other side compromise host resistance, thereby facilitating disease risk (Harvell et al., 1999). Hence, we need to extend the study on evolution of disease resistance to the interaction of host genotype, pathogen genotype and the environment (GxGxE) to be able to make realistic predictions about the evolutionary potential of the respective host population (Lafferty & Kuris, 1999). Despite the growing number of epizootics in the marine realm, studies addressing the evolutionary potential of host populations to pathogen infections in the light of projected rising temperatures are still rare.

The Pacific oyster *Crassostrea gigas* (Thunberg 1793) is a prominent aquaculture species and well‐suited model for studying disease dynamics (Le Roux, Wegner, & Polz, 2016) that has been repeatedly subjected to extensive mass mortalities during periods of high temperature (Samain, 2011). Oyster mass mortalities, usually referred to as summer mortality syndrome, affect all life stages and underlie a complex aetiology of biotic and abiotic factors, of which temperature and pathogen infection, especially by bacteria of the genus *Vibrio* or Ostreid herpesvirus (OsHV‐1), are the driving forces (Degremont, 2011; Dégremont, Ernande, Bédier, & Boudry, 2007; Fey et al., 2015; Garnier, Labreuche, Garcia, Robert, & Nicolas, 2007; Lemire et al., 2014; Petton et al., 2015; Saulnier et al., 2010; Travers, Boettcher Miller, Roque, & Friedman, 2015; Wendling & Wegner, 2013).

Pacific oysters, originating from Japan, have been deliberately introduced to many coastal areas worldwide for aquaculture purposes. In many cases, they escaped from the aquaculture sites and established stable populations in the wild (Ruesink et al., 2005). In the European Wadden Sea, two invasion waves have been reported. In the south, oysters had been imported from British Columbia to the Oosterschelde (Netherlands) between 1964 and 1982, from where they spread northwards. In the north, seed oysters have been regularly imported since 1986 from British hatcheries to an oyster farm off the island of Sylt (Germany) (Reise, 1998), from where they successfully spread north‐ and southwards. Based on haplotype frequencies, Southern oysters differ significantly from northern oysters, separating the entire Wadden Sea oyster population into two genetically distinct groups (Moehler, Wegner, Reise, & Jacobsen, 2011). Since 2003, southern and northern oysters now occur at a continuum along the entire coastline in the European Wadden Sea (Essink, Dankers, & Reise, 2005) and the two gene pools can now admix, which could lead to either a breakdown of evolved population‐specific GxGxE interactions (outbreeding depression) or overall higher resistance (hybrid vigour, Wendling & Wegner, 2015). While a scenario of admixture between two independent biological invasions might be comparatively rare, the frequent transport of oysters between localities in aquaculture (Muehlbauer et al., 2014) could have similar effects on GxGxE interactions in disease‐associated mass mortalities.

Disease resistance has a genetic basis in oysters (Dégrement, Morga, Trancart, & Pépin, 2016; Dégremont et al., 2005, 2007; Wendling & Wegner, 2015); however, variation underlying genotypic host x pathogen interactions as well as their environmental dependency has yet to be investigated. Therefore, the motivation of the present study was to determine the environmental modifications of GxG interactions against the background of two genetically distinct invasions that now form a secondary contact zone. By including an environmental component, we can thus extend the commonly observed GxG interactions between hosts and pathogens to GxGxE interactions and take a glance at the population‐specific evolutionary potential of Pacific oysters in changing and altered environments.

## 2 Material and methods

### 2.1 Admixture between invasive populations

**Figure 1:**
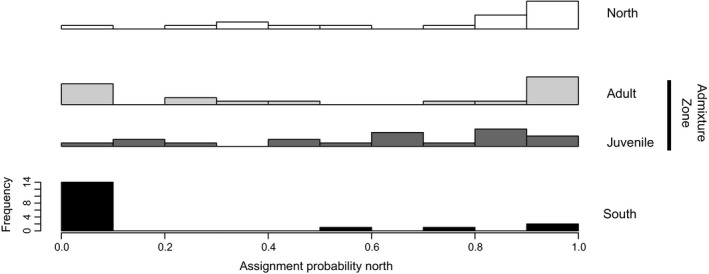
Probability of assignment to the northern cluster (left: 0%, right: 100%) based on the results of a Bayesian cluster analysis with *k* = 2 clusters for each group, that is, north (top), south (bottom), admixture zone_adults (middle, top), admixture zone_juveniles (middle, bottom)

To assess whether admixture occurs in the secondary contact zone, we analysed seven microsatellite loci (Li, Hubert, Bucklin, Ribes, & Hedgecock, 2003) of 20 Pacific oysters from both invasion waves, that is, north (Sylt: 55°2.33′N, 8°26.57′E) and south (Texel: 53°08.85′N, 4°54.53′E; Figure 1). Additionally, we included 20 adult and 20 juvenile oysters (average shell length <50 mm) from the potential admixture zone (Husum: 8°58.04′E, 55°2.33′N).

DNA from mantel tissue was extracted using a 96‐well DNA extraction kit (Wizard^®^Genomic DNA Purification Kit, Promega, Mannheim, Germany) following the manufacturer's protocol. PCR amplification was performed using the M13‐tailed PCR method (Schuelke, 2000) in a final reaction volume of 20 μl containing 4 μl Buffer (5×Colorless GoTaq^®^Flexi Buffer, Promega, Mannheim, Germany), 1.2 μl MgCl_2_ (25 mM), 0.4 μl dNTPs (10 mM), 0.04 μl labelled M13 primer (20 μM), 0.05 μl forward primer (20 pM/μl), 0.1 μl *Taq* polymerase (5 U/μl, GoTaq^®^DNA Polymerase, Promega, Mannheim, Germany) and 4 μl template DNA (approximately 10–20 ng/μl). Reactions were performed according to the following cycling protocol: 5 min at 94°C, 30 cycles of 30 s at 94°C, 45 s at 57°C and 45 s at 72°C, followed by 10 cycles of 30 s at 94°C, 45 s at 53°C and 45 s at 72°C, and a final extension at 72°C for 10 min. We denaturated 1 μl of each PCR reaction in HiDi‐formamide (Life Sciences, Darmstadt, Germany) and analysed it on an ABI prism 3100XL capillary sequencer (Life Sciences, Darmstadt, Germany) with GeneScan^®^ 500 LIZ^®^ as a size standard.

Microsatellite genotypes were scored using GeneMarker^®^ (Soft Genetics, version 1.91). After confirming that the most probable number of clusters (Evanno, Regnaut, & Goudet, 2005) reflected the separation of northern and southern invasions, assignment probability to the northern or southern population was calculated using a Bayesian cluster analysis implemented in the software STRUCTURE 2.2.3 (Pritchard, Stephens, & Donnelly, 2000).

### 2.2 Mating design, crosses and nursery protocol

Mature adult Pacific oysters were randomly collected from the northern and southern end of the Wadden Sea, that is, Texel (53°08.85′N, 4°54.53′E) representing the southern population and Sylt (55°2.33′N, 8°26.57′E) representing the northern population (Moehler et al., 2011). Full‐sib crosses were set up over a 2‐day period in July 2012 forming four groups with 10 families per group: two within‐population crosses, NN (North_female_ × North_male_) and SS (South_female_ × South_male_), and the two between‐population crosses, NS (North_female_ × South_male_) and SN (South_female_ × North_male_)_._ We produced reciprocal crosses to partition out the influences of different maternal and paternal life histories. Gametes were stripped directly from the gonads and collected into 0.45 μm filtered, UV‐treated seawater. Fertilization was performed at a ratio of 200 spermatozoa per oocyte, with 4 × 10^5^ oocytes per family. After 20 min, spermatozoa were removed by collecting oocytes on a 20‐μm mesh screen. One hour after fertilization embryos were transferred to rearing tanks at a concentration of five embryos/ml. Larvae were kept at 21°C with salinity at 28 psu in 2‐L rearing tanks filled with 0.45 μm filtered, UV‐treated seawater and fed *Isochrysis galbana* at a concentration of 10–150 cells/μl depending on age. Water was exchanged every second day. After 3 days, we lost four families, 1 of group SS and NN, and 2 of group SN, leaving 36 families for later analyses.

### 2.3 Bacterial isolates

We chose two representatives of the *Vibrio splendidus* clade: *O7w_July* and *Tx5.1* (Thieltges, Engelsma, Wendling, & Wegner, 2013) for the present infection experiment because they both showed similar degrees in virulence, induced relatively low levels of mortality at 17°C and showed consistent patterns of virulence over time (Wendling & Wegner, 2013). Strains inducing higher levels of mortality were not suitable because too high mortality rates would be expected, making it difficult to assess differences in survival.

Selected *Vibrio sp*. strains were grown under agitation at 25°C in nutrient solution 1.5% NaCl (1,000 ml distilled water, 5.0 g peptone, 3.0 g meat extract) for 20 hr. We evaluated the bacterial concentration by optical density at 550 nm representing a concentration of 5 × 10^8 ^CFU/ml in both strains (Gueguen et al., 2003; Wendling & Wegner, 2015). Bacteria cells were centrifuged at 5, 400 g at 25°C for 5 min and resuspended in nutrient solution at 2 × 10^9^ cells per ml.

### 2.4 Experimental design

To detect differences between different life stages in resistance of host genotype to sympatric and allopatric *Vibrio sp*. infection and the influence of temperature on such genotype x genotype interactions, we conducted two controlled infection experiments. The first experiment was carried out on D‐Larvae, that is, 7 days posthatching, and the second on Pediveliger larvae, that is, 18 days posthatching. We used a three‐way factorial design, with origin, temperature and infection as fixed factors. “Infection” had three levels (*O7w_July* (*Vibrio* Sylt) and *Tx5.1* (*Vibrio* Texel), respectively as well as PBS as control), “cross‐type” had three levels (NN, SS and hybrid) and “temperature” had two levels (19°C representing the present water temperature at which spawning occurs naturally and 23°C representing predicted future water temperature during summer months).

### 2.5 Experimental challenge

Controlled infection experiments on larvae were carried out using sterile 96‐well culture plates as described in Wendling, Batista, and Wegner (2014). Briefly, 10–15 larvae per family were placed in one micro‐well containing 0.45 μm filtered, UV‐treated seawater. Larvae were bath challenged with the selected *Vibrio* isolates at a concentration of 10^7 ^cells/ml, and each experimental group was replicated twice. To avoid the influence of potential room effects (Rohr et al., 2011), we placed the 96‐well plates in water baths separated by styrofoam that were set to either 19 or 23°C. Survival was measured 3 days postinfection using an inverted microscope by counting the amount of dead larvae, that is, closed larvae without internal movement.

In addition to survival, we measured the size of 20–50 larvae prior to both infection experiments. Larvae were fixed in Baker's formol‐calcium fixative (4% formaldehyde, 2% NaCl, 1% calcium actetate) (Wootton & Pipe, 2003) and photographed (Canon EOS 500D) under a microscope (Leitz Aristoplan) for subsequent digital size analysis using Leika QWin software.

### 2.6 Data analysis

All statistical analyses were performed in the R 2.15.2 statistical language (R Core Team, 2015).

The proportions of surviving larvae in each well were arc sin square root transformed. There was no significant difference in mean larval size between the different hybrid cross‐types at both stages (linear model: D‐larvae: *F*_(df=2,32)_ = 1.04, *p* = .36 and Pediveliger larvae: *F*_(df=2,31)_ = 0.20, *p* = .82). Similarly, we did not detect a significant difference in survival rate between NS and SN families at both larval stages (linear model: D‐Larvae: *F*_(df=1,214)_ = 0.22, *p* = .65 and Pediveliger larvae: *F*_(df=1,214)_ = 0.27, *p* = .6). We can therefore rule out population‐specific maternal and paternal effects and subsequently pooled the results of both between‐population crosses for the main analyses. Addition of both *Vibrio* strains had a significant influence on survival at both larval stages (linear model: D‐Larvae: *F*_(df=1,429)_ = 505.2, *p *< .001 and Pediveliger larvae: *F*_(df=1,429)_ = 334.6, *p *< .001, Figure 1). To test for GxG interactions, we excluded the control group from the main analysis.

Larval survival rate was analysed using a linear mixed‐effects model with maximum‐likelihood error estimation using the lme function (package nlme) with larval stage, temperature, cross‐type and pathogen genotype as well as all interactions as fixed effect and host (full‐sib family) as random effect. We observed that larval stage explained most of the variation in survival rate (*F*_(df=1)_ = 35.14, *p *< .001). In addition, at D‐larvae stage, survival was significantly correlated with size (linear model: *F*_(df=1,261)_ = 27.73, *p *< .001) and, however, not at Pediveliger stage (linear model: *F*_(df=1,261)_ = 0.42, *p* = .52). We therefore used separate linear models on the residuals of the correlation between survival and size to estimate the effect of temperature, pathogen genotype, host genotype and cross‐type on larval survival at each larval stage.

In addition, we studied family‐based thermal reaction norms for all cross‐types, pathogen genotypes and larval stages. We calculated the slope of the reaction norm for each family based on the net difference in survival between 19 and 23°C. Differences in reaction norm slopes were analysed using a linear mixed‐effects model with cross‐type, larval stage, pathogen genotype and all interactions as fixed effects and host genotype (full subfamily) as random effect.

## 3 **Results**

### 3.1 Admixture between invasive populations

Allele frequencies of the seven microsatellite loci showed a partitioning into *k* = 2 subgroups (Evanno et al., 2005), representing the southern and the northern population. The majority of oysters from north (55%) and south (80%) were assigned to their correct group with high probability (>90%). Adult oysters from the admixture zone consisted of a mixture of northern or southern individuals with high assignment probability (30% to cluster south and 40% to cluster north). Juvenile oysters from the admixture zone had significantly less individuals with high assignment probability to either cluster (χ^2^_df=1_ = 8.181, *p* = .004) and showed a shift towards lower assignment probabilities in general (Figure 1), which suggests ongoing admixture between both populations.

### 3.2 Infection experiment

**Figure 2:**
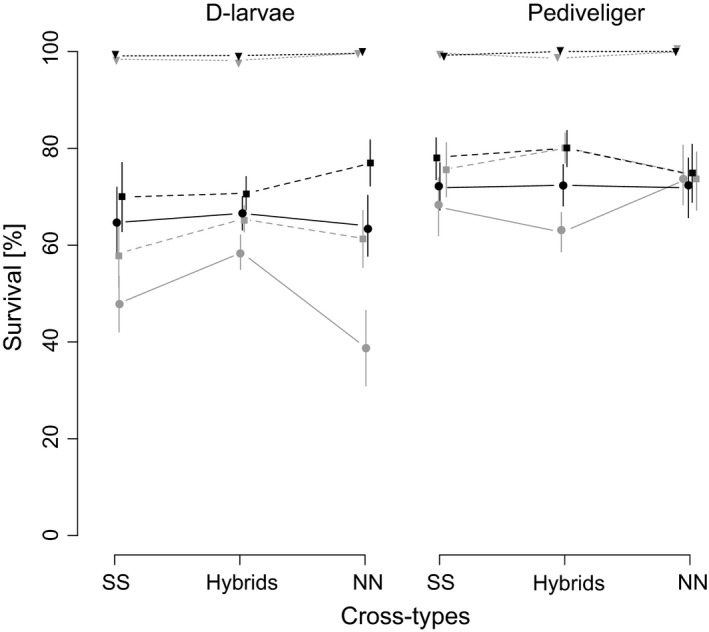
Survival rate of infected (solid: *Vibrio* north, dashed: *Vibrio* south) and control (dotted line) SS, hybrid and NN oysters at cold (grey, 19°C) and warm (black, 23°C) water temperatures, D‐larvae (left), Pediveliger larvae (right)

Larval stage, temperature, pathogen genotype and the interaction of larval stage and temperature significantly contributed to the observed variation in survival rate (Table 1). Survival rate was on average significantly higher in Pediveliger than in D‐larvae (linear model: *F*_(df=1,162)_ = 15.84, *p *< .001; Figure 2). We observed that host and pathogen genotype were significant main effects at both larval stages and thus conclude that there is a substantial genetic basis for disease resistance and that survival also depends on pathogen genotype (Table 2). Contrary to expectations, life stages differed significantly in their temperature tolerance upon *Vibrio* infection (Figure 2). While controlled infection experiments on adult oysters showed a reduced survival rate at high temperatures (Wendling & Wegner, 2013), we could not observe any influence of temperature on infection outcome in Pediveliger larvae and even a reverse temperature effect in D‐larvae (Figure 2). A significant interaction of temperature and host genotype as well as of pathogen genotype and host genotype at D‐stage larvae indicated a family‐specific response to temperature and infection. At D‐stage larvae, the net effect of temperature explained most of the observed variation in early larval stage survival (Table 2), and hence, the interaction of temperature as well as host and pathogen genotype (GxGxE) was only significant at D‐stage larvae. In contrast, at Pediveliger stage, survival was independent of temperature and only affected by host and pathogen genotype, as well as their interaction (GxG).

**Table 1:** Linear mixed‐effects model for survival with larval stage (stage), temperature (E), pathogen genotype (PG), cross‐type and all interactions as fixed effect and host genotype (full‐sib family) as random effect. Significant values are highlighted in bold

Fixed factor	numDF	denDF	*F*	*p*
(Intercept)	1	518	1,433.6335	<.001
**Stage**	**1**	518	**35.1369**	**<.001**
E	1	518	**16.6009**	**.0001**
PG	1	518	**23.1331**	**<.001**
Cross‐Type	2	33	0.2714	.764
Stage*E	**1**	518	**6.1037**	**.0138**
Stage*PG	1	518	0.0369	.8477
E*PG	1	518	1.8166	.1783
Stage* Cross‐Type	2	518	0.8823	.4144
E*Cross‐Type	2	518	0.4829	.6173
PG*Cross‐Type	2	518	0.1103	.8956
Stage*E*PG	1	518	0.0013	.9712
Stage*E* Cross‐Type	2	518	2.466	.0859
Stage*PG* Cross‐Type	**2**	518	**3.3395**	**.0362**
E*PG* Cross‐Type	2	518	0.0666	.9356
Stage*E*PG* Cross‐Type	2	518	0.5936	.5527

**Table 2:** Linear models for survival rate at each larval stage and the effect of temperature (E), cross‐type, host and pathogen genotype (H_G_, P_G_) and their interactions. Significant values are highlighted in bold

Fixed factor	D‐Larvae				Pediveliger Larvae				Fixed factor
	DF	Sum Sq	*F*	*p*	DF	Sum Sq	*F*	*p*	
**E**	**1**	**1.77**	**58.91**	**<.001**	1	0.07	0.97	.33	E
**PG**	**1**	**0.87**	**29.02**	**<.001**	**1**	**0.94**	**12.52**	**<.001**	**PG**
**Cross‐Type**	**2**	**0.42**	**6.92**	**.001**	2	0.01	0.09	.92	Cross‐Type
**HG**	**30**	**8.49**	**9.44**	**<.001**	**30**	**5.66**	**2.52**	**<.001**	**HG**
E*PG	1	0.01	0.28	.59	1	0.18	2.4	.12	E*PG
E*Cross‐Type	**2**	**0.37**	**6.12**	**.003**	2	<0.001	<0.001	1.0	E*Cross‐Type
PG*Cross‐Type	**2**	**0.27**	**4.43**	**.01**	2	0.2	1.32	.27	PG*Cross‐Type
E*HG	**30**	**2.39**	**2.65**	**<.001**	30	2.35	1.05	.41	E*HG
PG*HG	30	1.04	1.16	.27	**30**	**3.53**	**1.58**	**.04**	PG*HG
E*PG*Cross‐Type	2	0.03	0.46	.63	2	0.10	0.67	.51	E*PG*Cross‐Type
E*PG*HG	**30**	**2.11**	**2.34**	**<.001**	30	2.19	0.98	.51	E*PG*HG
Residuals	131	3.93			131	9.78			Residuals

When looking at the thermal reaction norms for survival in the three different cross‐types based on family level, we observed strong variation between families indicating a substantial amount of genetic variation within populations (Figs S1 and S2).

**Figure 3:**
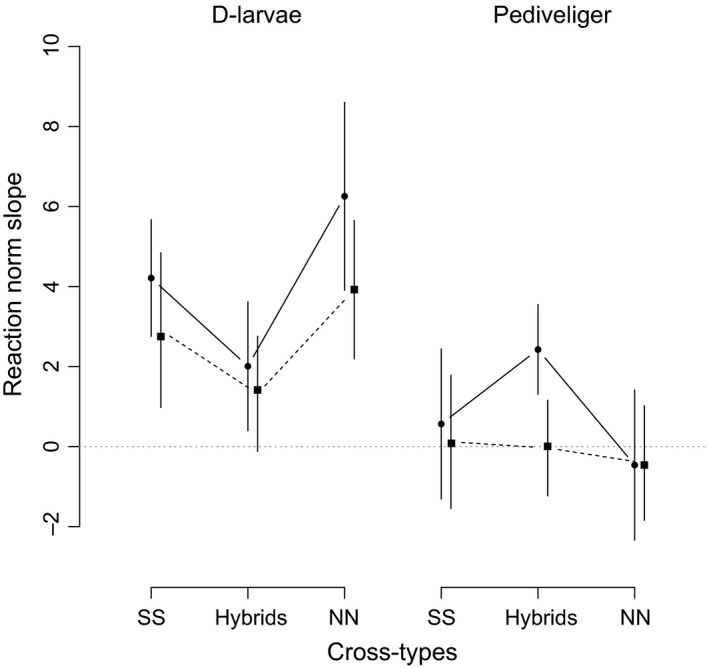
Mean slope of the reaction norms per cross‐type and infection for each larval stage based on the net difference in survival between 19°C and 23°C. Left side: D‐larvae, right side: Pediveliger larvae. Solid line: *Vibrio* north, dashed line: *Vibrio* south

Furthermore, we calculated the mean slope of the reaction norms per cross‐type and infection for each larval stage based on the net difference in survival between environments (Figure 3). Overall, we observed significantly steeper reaction norms in D‐larvae than in Pediveliger larvae (linear mixed‐effects model, larval stage effect *F*_(df=1)_ = 9.16, *p* = .003), reflecting the significant GxE interaction observed in D‐larvae, but absent in Pediveliger larvae. In D‐larvae, we also observed a trend for shallower reaction norms in hybrid crosses when compared against both pure cross‐types (cross‐type_SxS,TxT,H_ × stage interaction *F*_(df=2)_ = 2.642, *p* = .075) that were significant when combined pure cross‐types were contrasted against the hybrids (cross‐type_Pure,H_ × stage interaction *F*_(df=1)_ = 4.537, *p* = .036). This might indicate that the GxE interactions harbour a population‐specific component and can potentially cancel each other out when mating was performed between the populations.

## 4 Discussion

Disease is among the major causes of recent animal mass mortalities (Fey et al., 2015), and their increase has been linked, on the one hand, to rising temperatures (Harvell et al., 1999; Harvell et al., 2002) but on the other hand to host genotypes, as well as their interaction with the environment (GxE) (Thomas & Blanford, 2003). While such genotype by environment interactions are considered in many studies addressing the evolution of disease resistance, the additional influence of the pathogen and hence the three‐way interaction of hosts, pathogens and environment (GxGxE) has received far less attention. Using controlled infection experiments on offsprings from a full‐sib breeding design, we determined differences in resistance to oyster pathogens at present and future water temperatures within and between genetically differentiated oyster populations. Family and hence host genotype played a major role in disease resistance, but variation in disease resistance also depended on pathogen genotype as well as the interaction between host and pathogen genotypes (GxG). Interestingly, only in early life stages (D‐larvae), we could detect a significant environment effect on disease resistance in general as well as on the specific GxG interaction, which indicates that interactions between host, pathogen and the environment (GxGxE) determine the outcome of infections in very early larval stages. These population‐specific reaction norms were lost in hybrid crosses between both populations, showing that admixture between genetically differentiated stocks can influence GxGxE interactions on larger scales.

### 4.1 GxG and GxGxE interactions

In the present study, a substantial amount of variation in disease resistance is attributable to host families (i.e., genotypic units). Although full‐sib families contain maternal and paternal effects next to additive genetic variation, they nevertheless represent the reproductive unit that selection acts on in nature. Because we did not observe any population‐specific maternal effects, it seems fair to assume that disease resistance has a genetic basis, as previously shown (Wendling & Wegner, 2015). We could already show that host population affiliation plays a major role in explaining the variability for disease resistance in larval Pacific oysters reflecting a fast adaptive response to local pathogens (Wendling & Wegner, 2015). The dominant inheritance of resistance suggested a simple genetic basis and hence a high heritability for this trait. Here, we could now also show that overall population‐specific resistance patterns (Wendling & Wegner, 2015) can be modulated by a large variety of GxGxE interactions among families. Because we also used the same bacterial isolates for infections of adult oysters (Wendling & Wegner, 2015), our results here further suggest that the genetic variation in resistance to *Vibrio* infection is present in larval and adult life stages. A similar observation has been made for a genetically determined disease resistance across all several life stages of Pacific oysters to OsHV‐1 (Dégrement et al., 2016; Dégremont, 2013).

The outcome of an infection often depends on the interaction of host with pathogen genotypes (Lambrechts, Fellous, & Koella, 2006). Besides a significant family effect explaining some of the variability in disease resistance in Pacific oysters, we also provide evidence that susceptibility depends on the pathogen genotype, as well as on the interaction of host with pathogen genotypes (GxG).

Pacific oysters are considered as an important aquaculture species with estimated annual crop losses of more than 50% during mortality episodes (Cheney, MacDonald, & Elston, 2000). We are not aware of any study on oysters, where a significant GxG interaction has been detected. This is a major knowledge gap in studying disease resistance for such an important aquaculture species, as the evolution of resistance depends on both the host and the pathogen genotype (Lambrechts et al., 2006). In *Vibrio*‐associated diseases of oysters, the unit of pathogenesis is often a collection of clones forming a coherent bacterial population (Le Roux et al., 2016; Lemire et al., 2014) and oysters are usually colonized by a large diversity of *Vibrios* (Wendling et al., 2014). The large variation in resistance based on strong GxG interactions observed here might therefore reflect a selective response to such diversity of infection. Given that selection coefficients exerted by disease can be quite high, it is surprising that large amounts of genetic variation are maintained in affected populations. The scaling of phenotypic change with time strongly suggests that fluctuating selection pressures can explain the maintenance of genetic variation (Messer, Ellner, & Hairston, 2016), especially when selection pressures vary across life stages and generations overlap (Ellner & Hairston, 1994). The different responses of early life stages of oyster larvae observed here support these predictions, and when coupled to the dynamic nature of *Vibrio* community change, the genetic variation of plastic reaction norms might be an efficient response to such fluctuating selection pressures.

In the marine realm, most pathogens originate from warm water bodies or are correlated with high temperatures (Harvell et al., 1999). Due to its semi‐enclosed character, the North Sea is suggested to be very vulnerable to rising temperatures (Belkin, 2009). Therefore, we included a temperature component to study the evolution of disease resistance in Pacific oysters. Based on family‐based thermal reaction norms, we could show variable survival between host and genotypes upon pathogen infection across temperatures, indicating a high amount of phenotypic plasticity. The recent availability of the *C. gigas* genome found a large diversity of genes enabling oysters to cope with environmental stressors, for instance fluctuations in temperature or salinity (Zhang 2012). In particular, thermal tolerance has been shown to be very plastic and to correlate with changes in the expression of one family of heat shock proteins (Hamdoun 2003). Thus, high phenotypic plasticity, as observed in the present study, might help oysters to respond quickly to environmental alterations, such as temperature fluctuations.

Interestingly, a significant influence of temperature on disease outcome as well as on the interaction between host genotype, pathogen genotype and environment (GxGxE) could only be observed at very early larval stages. We could previously show that rising temperatures trigger mortality upon *Vibrio* infection in adult oysters (Wendling & Wegner, 2013; Wendling & Wegner, 2015). However, the present results point in the opposite direction and contrast several studies demonstrating a direct link between *Vibrio* virulence and increasing temperatures (Kimes et al., 2012; Mahoney, Gerding, Jones, & Whistler, 2010), as well as many theories addressing the interaction of rising temperature with emerging marine diseases in general (Harvell, Altizer, Cattadori, Harrington, & Weil, 2009; Harvell et al., 1999, 2002). Because our results here are based on two closely related *Vibrio* isolates that showed no population‐specific virulence patterns, the observed GxGxE interaction might show a pattern typical for this specific group of *Vibrios*. This might therefore reflect the specific temperature preferences of these strains rather than a general pattern observed for the majority of *Vibrio* strains. To address this issue, similar experiments as presented here have to be performed investigating GxGxE interactions on a larger variety of *Vibrio* spp. isolates.

### 4.2 Effect of temperature on life stage‐dependent disease resistance

In general, Pacific oysters have a wide temperature tolerance as has been shown for adults (Sicard et al., 2006) and larvae (Rico‐Villa, Pouvreau, & Robert, 2009). However, empirical studies demonstrated that larvae are dependent on temperatures above 22°C to reach optimal growth and metamorphosis performance while temperatures as low as 17°C consistently inhibit ingestion and growth over the entire larval period, although they did not cause significant mortalities (Rico‐Villa et al., 2009). Increased survival rate at 23°C in contrast to 19°C as shown in the present study could therefore result from a reduced physiological performance of early larval stages at colder temperatures resulting in impaired defence mechanisms against pathogens. As we did not observe any difference in susceptibility in control oysters between cold and warm waters, we assume that the lower experimental temperature is not harmful in general, but only when pathogens or other stressors are present. Interestingly, this temperature‐dependent disease resistance disappeared at the Pediveliger stage. In particular, Pacific oyster larvae are hypothesized to have a strong innate immune system that has a faster maturation rate in contrast to other marine bivalves (Luna‐Gonzalez, Maeda‐Martinez, Sainz, & Ascencio‐Valle, 2002). Thus, we hypothesize that in Pediveliger larvae the maturation of the immune system has reached a stage where temperature has no significant effect on defence capabilities anymore. Similar conclusions come from a study on resistance to ostreid herpesvirus (OsHV‐1), where it has been shown that resistance to mortality caused by OsHV‐1 increased with age and size, suggesting an age‐ and size‐dependent maturation of the immune system against the virus (Dégremont, 2013).

In species with type‐III survivor curves like oysters, early life stages are more vulnerable. Accordingly, we found lower survival rates in D‐larvae and only here survival correlated positively with size, indicating that elevated growth rates allow a faster escape from vulnerable stages of the life cycle. The observed life stage‐dependent correlation between size and survival could at least partly be explained by a maturation effect of the immune system, as the primary cause for disease resistance in D‐larvae. We hypothesize that larger larvae have a better physiological status and can divert more resources to immune system maturation than smaller larvae and hence show better defence capabilities against pathogens. This effect may be absent at the Pediveliger stage, when there are presumably no differences in maturity of the immune system. However, we did not measure any defence capabilities or the physiological status of the challenged larvae and are therefore unable to tease apart the direct effects of temperature and larval size on the immune response in early life stages of Pacific oysters. One approach for the future would be to examine the defence mechanisms and physiology of the different life stages and size classes and to quantify the influence of temperature on larvae defence capability.

### 4.3 Effect of admixture between invasive populations on resistance reaction norms

Both fronts of the genetically differentiated invasion waves of the Pacific oyster in the Wadden Sea now overlap (Essink et al., 2005), and our data indicate ongoing admixture between both gene pools. Admixture can have profound effects on locally adapted traits and their interactions with the environment (Verhoeven, Macel, Wolfe, & Biere, 2011). However, only little is known about the effect of admixture on disease resistance, although this seems highly relevant for regularly transplanted aquaculture species like Pacific oysters (Muehlbauer et al., 2014) that can shape the genotypic composition of derived wild populations (Moehler et al., 2011). In Pediveliger larvae, the admixed families show more variation in survival than the source population and no coherent change in reaction norms. At the D‐veliger stage, on the other hand, the admixed hybrids showed less variation in survival at the D‐veliger stage than the source populations. This pattern translated into flatter reaction norms across temperatures, indicating that the pronounced GxGxE interactions at this life stage disappeared in admixed individuals. Flatter reaction norms can be interpreted as higher environmental tolerance, which would give admixed individuals an advantage in variable environments. On the other hand, this pattern can also be interpreted as a loss of population‐specific reaction norms that may reflect local adaptation. In general, admixed oysters should be more resistant against infection by a broad range of *Vibrio* strains due to dominant inheritance of resistance (Wendling & Wegner, 2015). The environmental component of this inheritance pattern remains however to be tested, and our data here might indicate that across environments populations‐specific reaction norms might be lost.

## 5 Conclusion and outlook

In this study, we detected a life stage‐dependent interaction of disease resistance and temperature. While infected adult oysters have been shown to be more susceptible at high temperatures (Wendling & Wegner, 2013; Wendling & Wegner, 2015), survival rates of infected early larval stages point in the opposite direction and are positively correlated with larval size. Early D‐larvae stages represented the more vulnerable life stage, and we can therefore assume that in the European Wadden Sea, invasive Pacific oysters are at their lower thermal limit. Oyster spawning and larval disease resistance are impaired at temperatures resembling average summer temperatures, that is, 19°C, but might be facilitated by increasing temperatures. However, when temperature will increase, as predicted by global climate change, adult and juvenile oysters will be more harmed by disease outbreaks, as has been shown in several coastal ecosystems worldwide (Cheney et al., 2000; Goulletquer et al., 1998; Imai, Numachi, Oizumi, & Sato, 1965; Perdue, Beattie, & Chew, 1981; Watermann et al., 2008) as well as in experimental studies (Wendling & Wegner, 2013). These two opposing processes must be offset against each other to derive population and evolutionary consequences for resistance against polymicrobial diseases (Le Roux et al., 2016).

In changing environmental conditions, the amount of standing genetic variation on which natural selection can act on to produce suitable phenotypes ultimately determines the persistence of species in the long term (Gienapp et al., 2008). If oyster mass mortalities arise from polymicrobial disease (Lemire et al., 2014; Petton et al., 2015), the maintenance of GxG interactions becomes of paramount importance. However, the survival of a single organism also depends on the amount of phenotypic plasticity to respond quickly to unfavourable conditions (Gienapp et al., 2008). Because admixture can influence these reaction norms, broodstock selection should also aim to maintain phenotypic plasticity in terms of temperature‐dependent disease resistance of different life stages within broodstocks to ensure a fast acclimatization potential. Similarly, the impact of aquaculture activities such as farming and transport on wild populations (Muehlbauer et al., 2014) should consider this impact for farming practices and conservation to respond to challenges of increasing frequencies of mass mortalities (Fey et al., 2015).

## Data Archiving Statement

Primary data are available through the data repository Pangaea (doi: 10.1594/PANGAEA.868904).
