# Supplementary figures and images for: Population‐specific genotype x genotype x environment interactions in bacterial disease of early life stages of Pacific oyster larvae
Source: Evol Appl. 2017 Mar 9;10(4):338–47. doi: 10.1111/eva.12452 (PMC5367073; doi:10.1111/eva.12452)

**NN**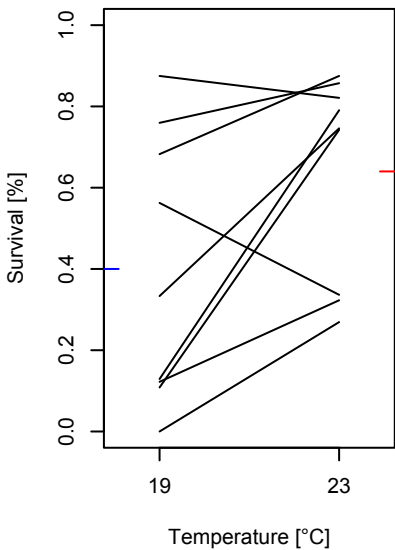**NH**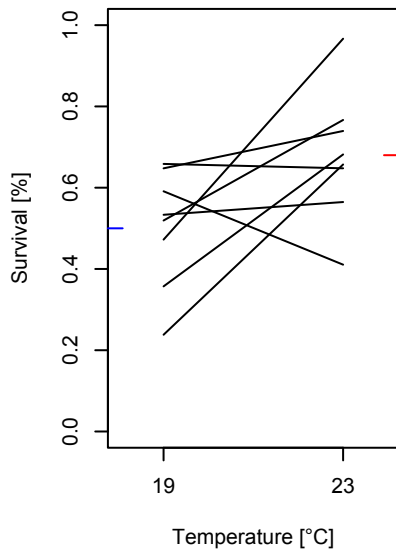**NS**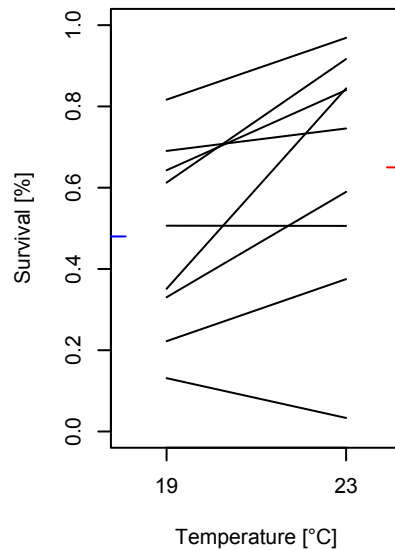**SN**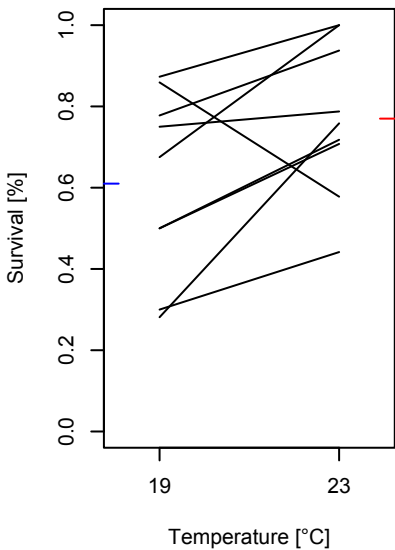**SH**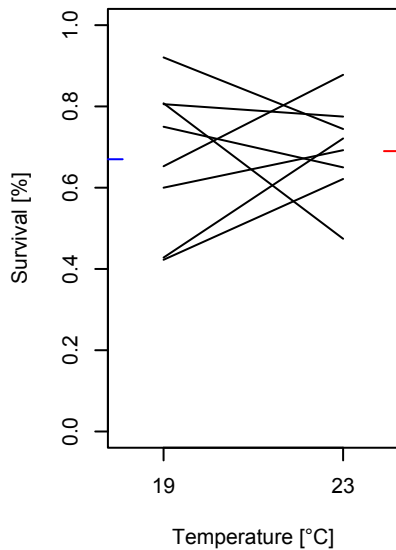**SS**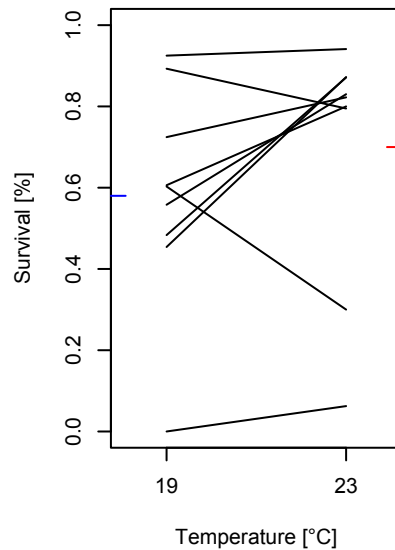

Supplement: Supplementary file 1 [file EVA-10-338-s001.pdf]

**NN**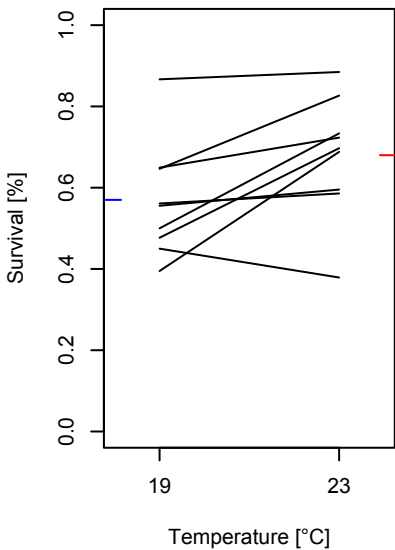**NH**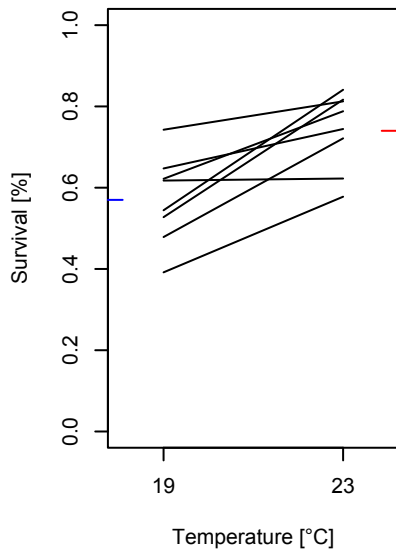**NS**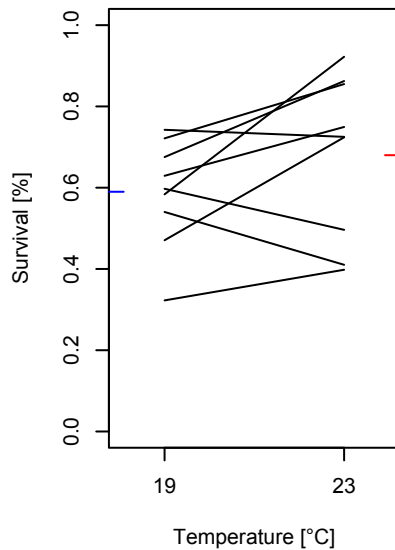**SN**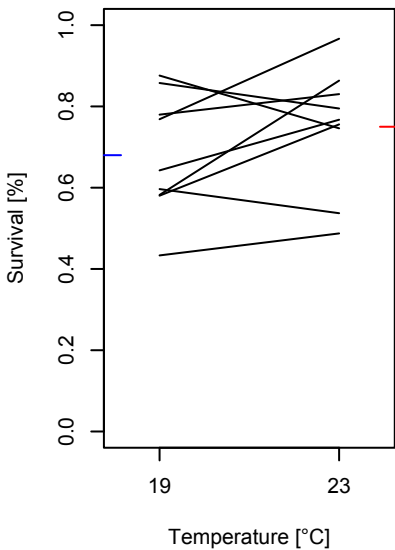**SH**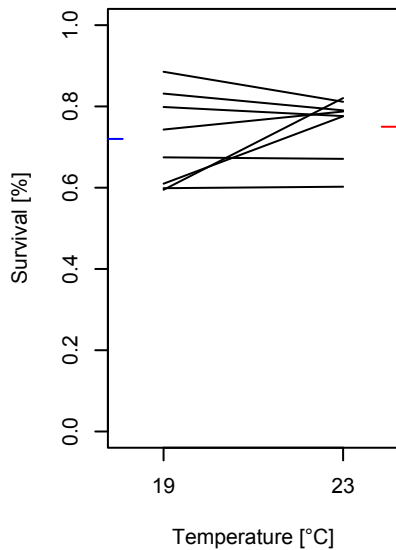**SS**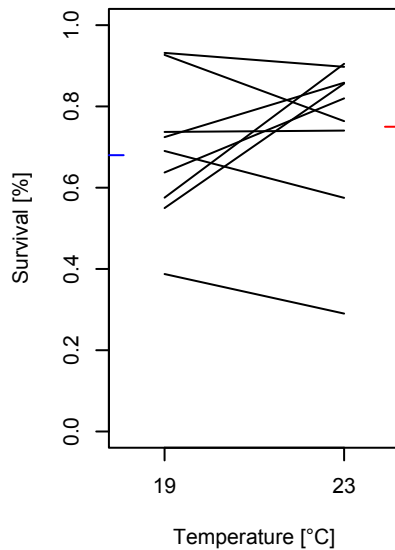

Supplement: Supplementary file 2 [file EVA-10-338-s002.pdf]
